# Targeted intervention of eIF4A1 inhibits EMT and metastasis of pancreatic cancer cells via c-MYC/miR-9 signaling

**DOI:** 10.1186/s12935-021-02390-0

**Published:** 2021-12-14

**Authors:** Yuchong Zhao, Yun Wang, Wei Chen, Shuya Bai, Wang Peng, Mengli Zheng, Yilei Yang, Bin Cheng, Zhou Luan

**Affiliations:** 1 Department of Gastroenterology and Hepatology, Tongji Hospital, Tongji Medical College, Huazhong University of Science and Technology, Jiefang Avenue No. 1095, Wuhan, 430030 China; 2 Departement of Hepatobiliary and Pancreatic Surgery, The First Affiliated Hospital of Zhengzhou University, Jianshe East Road No. 1, Zhengzhou, China

**Keywords:** Eukaryotic translation initiation factor 4A1, c-MYC, Epithelial-mesenchymal transition, Rocaglamide, Mycro3

## Abstract

**Background:**

Owing to the lack of effective treatment options, early metastasis remains the major cause of pancreatic ductal adenocarcinoma (PDAC) recurrence and mortality. However, the molecular mechanism of early metastasis is largely unknown. We characterized the function of eukaryotic translation initiation factors (eIFs) in epithelial-mesenchymal-transition (EMT) and metastasis in pancreatic cancer cells to investigate whether eIFs and downstream c-MYC affect EMT and metastasis by joint interference.

**Methods:**

We used The Cancer Genome Atlas (TCGA) and Genome Tissue Expression (GTEx) databases to analyze eIF4A1 expression in PDAC tissues and further validated the findings with a microarray containing 53 PDAC samples. Expression regulation and pharmacological inhibition of eIF4A1 and c-MYC were performed to determine their role in migration, invasion, and metastasis in pancreatic cancer cells in vitro and in vivo.

**Results:**

Elevated eIF4A1 expression was positively correlated with lymph node infiltration, tumor size, and indicated a poor prognosis. eIF4A1 decreased E-cadherin expression through the c-MYC/miR-9 axis. Loss of eIF4A1 and c-MYC decreased the EMT and metastasis capabilities of pancreatic cancer cells, whereas upregulation of eIF4A1 attenuated the inhibition of EMT and metastasis induced by c-MYC downregulation. Treatment with the eIF4A1 inhibitor rocaglamide (RocA) or the c-MYC inhibitor Mycro3 either alone or in combination significantly decreased the expression level of EMT markers in pancreatic cancer cells in vitro. However, the efficiency and safety of RocA alone were not inferior to those of the combination treatment in vivo.

**Conclusion:**

Overexpression of eIF4A1 downregulated E-cadherin expression through the c-MYC/miR-9 axis, which promoted EMT and metastasis of pancreatic cancer cells. Despite the potential feedback loop between eIF4A1 and c-MYC, RocA monotherapy is a promising treatment inhibiting eIF4A1-induced PDAC metastasis.

**Supplementary Information:**

The online version contains supplementary material available at 10.1186/s12935-021-02390-0.

## Introduction

Pancreatic ductal adenocarcinoma (PDAC) is universally one of the most lethal solid malignancies. Despite its relatively low incidence, PDAC remains the fourth leading cause of cancer-related death in developed countries [1, 2], with no significant changes in the mortality to incidence ratio in the past several decades and a five-year survival rate stagnating at approximately 3–15% [3]. At the early stage of carcinogenesis, pancreatic cancer cells can metastasize to distant organs through epithelial-mesenchymal transition (EMT) [4]. An overwhelming majority of patients diagnosed with PDAC have already lost the chance to undergo surgical resection due to early metastasis, which is also the key reason for postoperative recurrence. KRAS was the most commonly mutated oncogenes associated with PDAC [5]. To date, all attempts to target common PDAC KRAS variants (e.g., G12D, G12V, G12R) and KRAS downstream kinases (e.g., RAF, MEK, ERK, PI3K) have failed in phase I/II clinical trials [6–9]. Thus, novel therapeutics other than inhibitors targeting KRAS-associated kinases are urgently needed for PDAC patients.

Uncontrolled protein production is a common characteristic of cancer cells, and it is also necessary for EMT and metastasis [10]. Therefore, intervening to slow the hyperactive protein production has become a possible therapeutic strategy for PDAC. Translation initiation regulated by eukaryotic translation initiation factors (eIFs) is the most important rate-limiting step in translation [11, 12]. Dysregulation of eIF expression and function is a hallmark of various types of cancers including PDAC; the eIF4F heterotrimeric complex is the main factor facilitating mRNA translation. Meanwhile, eIF4F activity is largely regulated by RAS signaling, which further indicates that eIF could play an important role in PDAC [13, 14]. The eIF4F complex is composed of the scaffold protein eIF4G, cap-binding protein eIF4E, and ATP-dependent DEAD-box RNA helicase eIF4A. Previous studies typically targeted eIF4E to inhibit EMT and metastasis of cancer cells, because eIF4E is generally overexpressed in multiple cancers. However, recent studies demonstrated that there are eIF4E-independent and eIF4A-dependent binding sites for oncogenic mRNA downstream of eIFs, including c-MYC, which could explain why studies targeting eIF4E were unsuccessful [15, 16]. eIF4A is the only regulatory enzyme-catalytic factor in the eIF family, and facilitates unbinding the complex long-sequence helix (CLSH) in mRNA 5′-untranslated region (5′-UTR). The CLSH is a typical signature of various mRNAs downstream of eIFs, including c-MYC [17]. Overexpression of c-MYC is a carcinogenesis driver for multiple cancers and c-MYC is the most activated oncogenes [18, 19]. c-MYC is a crucial regulator of EMT and metastasis by promoting miR-9 expression [20, 21]. However, due to the structure of c-MYC, there are few c-MYC targeted inhibitors to dare [22, 23]. Considering that eIF4A is dispensable for c-MYC translation, we hypothesized that interfering with eIF4A function could be an effective way to inhibit c-MYC.

Traditional Chinese medicinal herbs have garnered increasing attention as a novel source of anticancer remedies within the past decade. Rocaglamide (RocA) is a cyclopenta-b-benzofuran-type compound derived from traditional Chinese remedies genus *Aglaia* (family *Meliaceae*). Iwasake et al*.* conducted a survey in 2016 and found that RocA could firmly bind eIF4A at the 5′-UTR sequence and suppress hyperactive protein production more effectively than eIF4E intervention [24]. Subsequently, RocA was used to treat various hematologic malignancies (e.g., myeloma and T-cell lymphoma) in a mouse model and exerted prominent antitumor efficacy without observed side effects [25]. Trials of RocA in solid malignancies are rare, one of which was our previous study demonstrating that RocA obviously repressed EMT and metastasis of pancreatic cancer cells in a mouse model [26]. Nevertheless, how eIF4A affects EMT and metastasis of PDAC is still largely unknown. Considering the complex feedback loop between eIF4A and c-MYC, whether simultaneous inhibition of eIF4A and c-MYC was superior to targeting either alone merits investigation. There are 3 identified isoforms eIF4A in humans. eIF4A1 is the major type that participates in the assembly of eIF4F in cancer cells. eIF4A2, which is mainly expressed in cells with low levels of proliferation, is correlated with a good prognosis in multiple cancers [12, 17]. And the regulatory role of eIF4A3 remains largely elusive in cancer cells [27]. Therefore, we selected eIF4A1 as the target in our experiments.

Here, we reported that eIF4A1overexpression downregulated the expression of E-cadherin through c-MYC/miR-9 signaling axis, which promoted EMT and metastasis.

## Materials and methods

### Cell culture and reagents

An immortalized pancreatic ductal epithelial cell line (HPDE) and pancreatic cancer cell line (Canpan-2) were obtained from the Cell Bank of the Chinese Academy of Sciences (Shanghai, China), and the pancreatic cancer cell lines AsPC-1, BxPC-3, and Panc-1 were purchased from the American Type Culture Collection (VA, USA). Cells were cultured in RPMI-1640 medium (GIBCO, NY, USA) supplemented with 10% fetal bovine serum (FBS, GIBCO) and 1% penicillin/streptomycin (Invitrogen) at 37 °C in a 5% CO_2_ incubator. The eIF4A inhibitor RocA and c-MYC inhibitor Mycro3 were purchased from MedChemExpress (MCE, USA).

### Patient’ follow-up and specimens collection

This study was approved by the Ethics Committee of Tongji Hospital of Tongji Medical College, Huazhong University of Science and Technology (TJ-IRB20210927). All patients were completely informed of the possible use of their clinical information/specimens and we obtained full consent for the study. The cohort comprised 53 patients with PDAC who underwent surgical resection from 2009 to 2013 at the Department of Pancreatic and Hepatobiliary Surgery, Tongji Hospital of Tongji Medical College, Huazhong University of Science and Technology (Wuhan, China). All PDAC specimens were verified by pathologists and met the criteria set by the American Pancreatic Association. None of the patients underwent preoperative neoadjuvant therapy. The patients were evaluated every 3 months during the first 3 years and every 6 months thereafter by physicians who were blinded to the study parameters. Follow-up data were summarized at the end of December 2020. To evaluate the prognostic role of eIF4A1, tissue microarrays of the 53 PDAC samples were collected for further analysis.

### Immunohistochemistry (IHC)

IHC staining with the eIF4A1 antibody (Abcam ab31217) was performed to detect the protein expression level. ImageJ (http://imagej.nih.gov/ij) IHC profiler (http://sourceforge.net/projects/ihcprofiler) was used to assess IHC staining of the microarray based on two categories: staining intensity and percentage of staining. The staining intensity was scored as follows: negative (0 points), weak-positive (1 point), positive (2 points), and strong-positive (4 points). The protein expression level was quantified by multiplying the staining intensity and corresponding extents of positive staining (n%: percentage of positive areas to the whole areas). Then we divided the patients into two groups (score < 50, low expression; score > 50, high expression) and performed subsequent survival analysis. The IHC staining results were reassessed by two independent pathologists who were blinded to this study.

### Immunofluorescence

Paraformaldehyde fixed samples were washed 3 times with ice-cold PBS for 3 min each time and then incubated in 10% donkey serum in PBS for 20 min. Subsequently, the samples were incubated with the eIF4A1 antibody (Abcam ab31217) in PBS at 4 °C overnight. After washing, fluorochrome-conjugated secondary antibodies (1:400, Alexa Fluor®488 donkey anti-rabbit IgG) were used, and then the samples were treated with DAPI. The fluorescence was visualized under an Olympus microscope.

### Lentiviral transduction

The lentivirus pLVX-Puro (Addgene) was obtained from DesignGene Biotechnology (Shanghai, China) and used to clone shRNA sequences. The vectors were designated Lv-eIF4A1 (eIF4A1-1, 5′-CACACTGGACTAGTGGATCCCGCCACCATGTCTGCGAGCCAGGATTCCC-3′; eIF4A1-2, 5′-AGTCACTTAAGCTTGGTACGATGAGGTCAGCAACATTGAGG-3′), Lv-sh-c-MYC (c-MYC-1, 5′-GCTTCACCAACAGGAACTATG-3′; c-MYC-2, 5′-GCTTGTACCTGCAGGATCTGA-3′; c-MYC-3, 5’-GGAAACGACGAGAACAGTTGA-3′) and Lv-sh-control (empty vector). The lentivirus plasmid and packaging plasmids were transfected into pancreatic cancer cells with transfection reagent (Lipofectamine®3000, Thermo Fisher Scientific) in OPTI-MEM media (Invitrogen, MA, USA). Lentiviral infection of the target cells was performed in cell culture medium containing 5 μg/ml polybrene (Sigma H9268), and infected cells were selected with 2.5 μg/ml puromycin for follow-up experiments.

### Quantitative real-time PCR (RT-qPCR)

cDNA was created with PrimeScript^TM^RT reagent Kit Perfect Real Time, (Takara, RR037A) according to the manufacturer’s protocol. Quantitative PCR was performed on a StepOne Real-Time System (Thermo Fisher Scientific) using TB Green® Premix EX Taq™ (Takara, RR820a) according to the manufacturer’s protocol. Melting curve analysis was performed and the amplification plots were evaluated by SDS 1.9.1 software (Applied Biosystems, MA, USA). The 2^−ΔΔCt^ method was used to determine relative fold changes in target gene expression from replicate samples. The bulge-loop primer for miR-9 was provided by RioBIO (Guangzhou, China).

### Western blot

Western blot analysis was performed as described previously [28]. The primary antibodies targeted eIF4A1 (Abcam ab31217), c-MYC (Abcam ab32072), snail (Abcam ab216347), E-cadherin (Abcam ab40772), andβ-actin (Abcam, ab8226). Protein bands were visualized using Beyo ECL Plus and quantified with ImageJ.

### Transient transfection

Cells were transinfected with scramble siRNA or siRNA targeting eIF4A1 (siRNA1, 5′-GAGTAACTGGAATGAGATT-3′; siRNA-2, 5′-TCCAGCAGCGAGCCATTC-3′; siRNA-3, 5′-CGTGTGTTTGATATGCTTA-3′) and c-MYC (siRNA-1, 5′-GAGGAGACATGGTGAACCA-3′; siRNA-2, 5′-GGGTCAAGTTGGACAGTGT-3′; siRNA-3, 5′-CGACGAGACCTTCATCAAA-3′) with Lipofectamine®3000 (Thermo Fisher Scientific) and OPTI-MEM (Invitrogen, MA, USA) according to the manufacturer’s protocol. eIF4A1-siRNA-1 and c-MYC-siRNA-3 were the most effective in knocking down their respective targets and were used for further analysis. Cells were transfected with CV567 empty vector, pcDNA3.1-eIF4A1 (P1, 5′-CACACTGGACTAGTGGATCCCGCCACCATGTCTGCGAGCCAGGATTCCC-3′) or pcDNA3.2-c-MYC plasmids (P1, 5′-CACACTGGACTAGTGGATCCCGCCACCATGGATTTTTTTCGGGTAGTGG-3′) using the transfection reagent Lipofectamine®3000 (Thermo Fisher Scientific) and OPTI-MEM (Invitrogen, MA, USA) according to the manufacturer’s protocol.

### In vitro migration and invasion assay

Cell migration and invasion were analyzed using Transwell chambers (8-μm pore size; Millipore, Billerica, MA, USA) with and without a Matrigel (BD Biosciences, San Jose, CA, USA) matrix in the upper chamber. Four groups of AsPC-1 cells were pretreated in DMSO, RocA (100 nM), Mycro3 (5000 nM), and combination treatment solutions for 4 h. Then the cells were seeded FBS-free culture medium in the upper chamber with 10% FBS culture medium in the lower chamber as a chemoattractant. After 28 h (Migration) or 32 h (Invasion) of incubation, cells on the lower surface of the membrane were washed with PBS, fixed in 4% methanol, and stained with a 0.4% crystal violet solution. Photographs of three randomly selected fields of the fixed cells were captured and cells were counted. The experiments were repeated independently three times.

### CCK8 assay

The sensitivity of cells to RocA and Mycro3 either or in combination was measured by CCK8 assay. Cells were seeded in a 96-well plate at density of 1000 cells per well and then treated with the treatment concentrations (RocA, 100 nM; Mycro3, 5000 nM). After the cells were attached. Then incubated for 4 h, replace fresh 1640 culture medium and cell counting kit-8 (CCK8, promoter, China) to each well according to the manufacturer’s protocol, incubated at 37 °C for 2 h. Absorbance was measured at 450 nm using a microplate reader (Thermo Scientific).

### Establishment of a metastatic mouse model

Animal experiments were approved by the Ethics Committee of Tongji Hospital of Tongji Medical College, Huazhong University of Science and Technology (TJH-202010007). Four-week-old female severe combined immune deficiency (SCID) mice (Charles River Co., Beijing) were maintained in a specific pathogen-free (SPF) environment. To establish the metastatic mouse model in vivo, 2 × 10^6^ AsPC-1 cells in 200 μl were intravenously injected via caudal vein into each animal. Small animal imaging (Spectral Imaging LagoX) was performed every 3 days after cell injection and the mice were randomly divided into different groups (5/ group). RocA (MCE, USA) was administered daily by intraperitoneal injection (5 mg/kg/d, 3 mg RocA dissolved in 30 μl DMSO, 600 μl PEG300 and 75 μl Tween-80 successively, and then adjusted to a volume of 1.5 ml with normal saline) every day originally [26], then adjusted to 2.5 mg/kg/d once on an alternate day. Mycro3 was administered intragastrically (100 mg/kg/d, 25 mg mycro3 dissolved in 2.5 ml of 0.5% methylcellulose solution and subject to ultrasonication) [29]. Control mice were treated with intragastric 200 μl of methylcellulose solution daily and 100 μl of methylcellulose solution via intraperitoneal injection once on an alternate day. When the total body weight loss was > 20%, the mice were sacrificed by cervical vertebra dislocation under deep sedation conditions using 70 mg/kg pentobarbital sodium (peritoneal injection).

### Subcutaneous xenografts in nude mice

Four-week-old nude mice were obtained from HFK Bioscience Ltd (Beijing, China) and maintained in SPF conditions. AsPC-1 cells (5.0 × 10^6^) suspended in a 100 μl solution comprising equal volumes of medium and matrix gel were subcutaneously implanted into the right flanks of 6-week-old female nude mice. When the tumors had reached a volume of approximately 60–90 mm^3^, the mice were then randomly divided into two groups. The treatment group received an intraperitoneal injection of RocA (previously adjusted dose), whereas the control group received cosolvent injection alone (n = 4, the treatments were carried out once daily for 28 days. The tumor volumes and total body weight of the animals were measured twice a week. The tumor volumes (mm^3^) were calculated with the following formula: V = LS^2^/2 (where L is the longest diameter and S is the shortest diameter). At the end of the experiment, the mice were sacrificed and the tumors were harvested for western blot analysis. The mice were sacrificed 4 weeks after implantation by cervical dislocation under deep sedation conditions using 70 mg/kg pentobarbital sodium (peritoneal injection).

### Statistical analysis

The data are representative of at least three independent experiments or multiple independent mice as indicated. The patient characteristics were summarized as the mean ± standard deviation (SD) for normally distributed continuous variables, median with interquartile range for continuous variables with a skewed distribution, and frequency (percentage) for categorical variables. All analyses were performed using R (http://www.R-project. org, version 3.5.2) with a two-sided significance threshold of *P* < 0.05.

## Results

### eIF4A1 was highly expressed in PDAC and predicted a poor prognosis

**Fig. 1:**
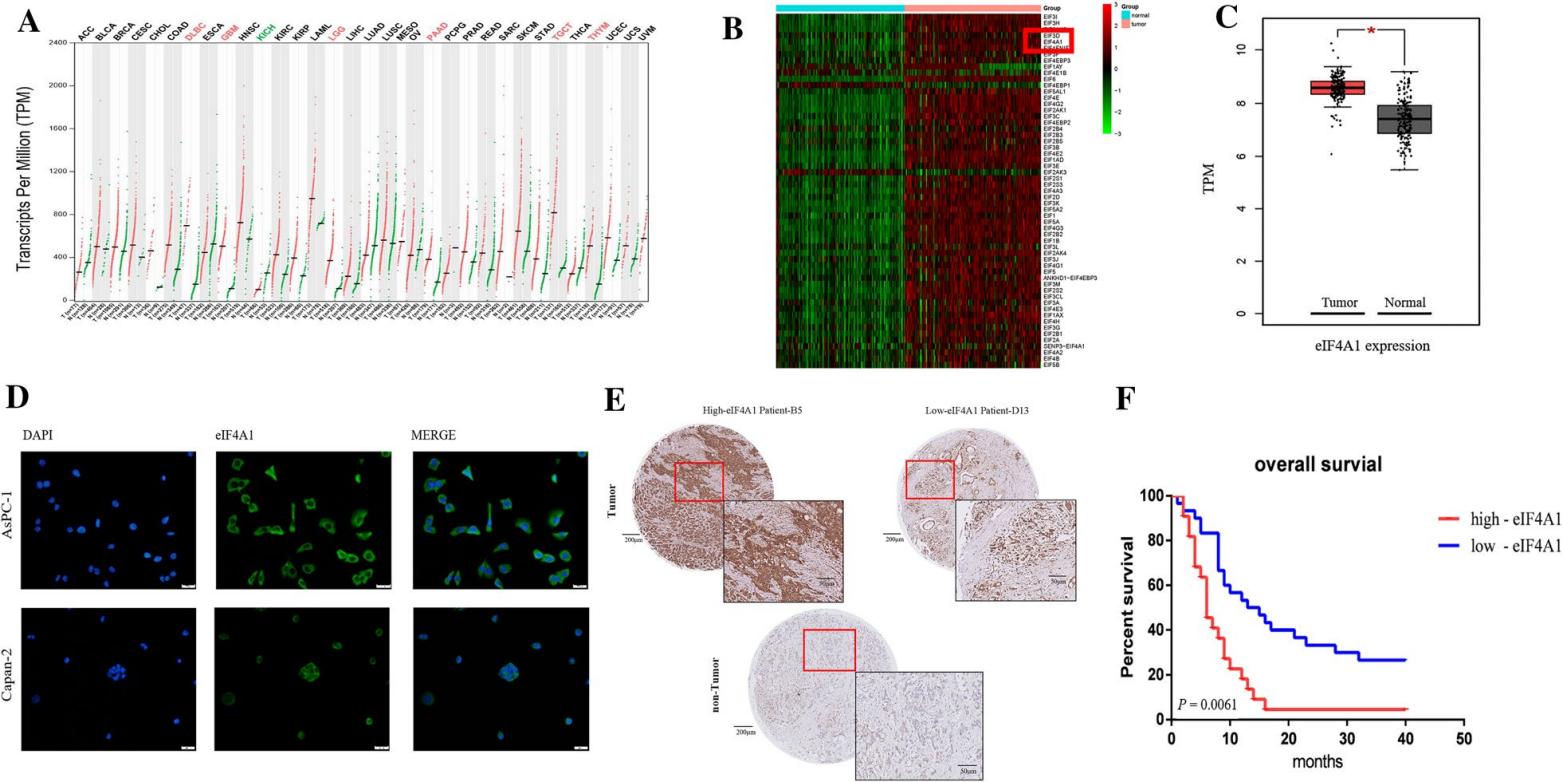
Analysis of the correlation of eIF4A1 expression and clinical characteristics of patients. **a** eIF4A1 expression was upregulated in various cancer types including pancreatic adenocarcinoma; **b** eIF4A1 was one of the top-upregulated genes of the protein synthesis-related eIF family in PDAC compared to healthy pancreatic tissues; **c** eIF4A1 was significantly overexpressed in PDAC tissues, log_2_[FC] = 1.162, P = 2.22e−16; **d** Immunofluorescence image of eIF4A1 localization (green) in pancreatic cancer cells (AsPC-1, Capan-2). eIF4A1 was predominantly located in the cytoplasm; **e** Representative images of IHC staining for eIF4A1 in a microarray comprising tumor and adjacent nontumor tissues, Scale bars: 100 μm. **f** Analysis of overall survival was compared according to the expression levels of eIF4A1 in PDAC tissues. Patients with high eIF4A1 expression had shorter overall survival (6 months vs. 9 months, log-rank test, HR = 2.096, 95% CI: 1.438–5.242, *P* = 0.0061)

First, we analyzed eIF4A1 expression across multiple cancers using data from Gene Expression Profiling Interactive Analysis (GEPIA, http://gepia.cancer-pku.cn/). eIF4A1 was highly expressed in multiple cancer types, including pancreatic adenocarcinoma, thymoma, glioblastoma multiforme, diffuse large B cell lymphoma, and testicular germ cell tumors (Fig. 1a). To evaluate the expression of all eIF family members in pancreatic adenocarcinoma, we analyzed the RNA-seq data from the TCGA and GTEx databases. Two samples were deleted after data quality control, resulting in 179 pancreatic tumor tissues and 169 normal pancreatic tissues. We ranked the differential expression of all eIFs (Fig. 1b) and the results showed that the expression level of eIF4A1 in pancreatic tumor tissues was significantly higher than that in normal tissues (Fig. 1c).

Subsequently, we analyzed the prognostic role of eIF4A1 in PDAC patients. Immunofluorescence results indicated that eIF4A1 was mainly localized in the cytosol (Fig. 1d), which was consistent with its function in assisting translation initiation. The fluorescence intensity in the more aggressive AsPC-1 cell line was significantly higher than that in Capan-2 cells. We used IHC to detect eIF4A1 expression in a tissue microarray comprising samples from 53 patients with pathologically confirmed PDAC from 2009 (Fig. 1e). The results indicated that patients with high eIF4A1 expression had a poor prognosis, and the median overall survival of patients with high eIF4A1 expression was significantly shorter than that of patients with low eIF4A1 expression (6.0 months vs. 9.0 months, HR = 2.10, 95% CI: 1.44–5.24, P = 0.0061) (Fig. 1f). Furthermore, we examined the correlations between eIF4A1 expression and multiple clinical features (Table 1, *P < 0.05; chi-square or Fisher’s exact test). Importantly, we found that a high level of eIF4A1 expression was significantly correlated with tumor size and lymph node metastasis. In summary, these results showed that eIF4A1 was highly expressed in pancreatic adenocarcinoma tissues, and high expression of eIF4A1 suggested a poor prognosis. The eIF4A1 expression was positively correlated with lymph node metastasis which is a major mechanism of cancer cell metastasis.

**Table 1:** Correlation between eIF4A1 expression and clinical characteristics of PDAC patients

Clinical characteristics	High-eIF4A1	Low-eIF4A1	*P*-value
Age, mean ± SD, years	66.04 ± 9.69	64.10 ± 10.07	0.482
Male, n (%)	13 (56.53%)	21 (70.00%)	0.311
Overall survival, median, months	6.0 (4.0, 9.0)	9.0 (8.0, 15.5)	0.006^b^
Differentiation status			0.523
Well differentiated	11 (20.75%)	17 (32.08%)	
Moderately to poorly differentiated	12 (22.64%)	13 (24.53%)	
Tumor size, mean ± SD, cm	4.57 ± 1.40	3.75 ± 1.28	0.032^b^
Recurrences, n (%)	17 (73.91%)	20 (66.67%)	0.569
Location			0.267
Head, n (%)	15 (28.30%)	18 (33.96%)	
Body/tail, n (%)	7 (13.21%)	20 (37.74%)	
Diffusion involvement, n (%)	1 (1.89%)	2 (3.77%)	
TNM stage^a^			0.077
I–II stage, n (%)	14	24	
III–IV stage, n (%)	9	5	
Lymph node metastasis, n (%)	14 (63.64%)	10 (35.71%)	0.0498*
Vascular Infiltration, n (%)	10 (43.48%)	3 (10.00%)	0.272

^a^NCCN Version 3.2019 pancreatic adenocarcinoma

^b^*P* < 0.05, significant difference

### eIF4A1 targeted c-MYC to regulate metastasis in pancreatic cancer cells

**Fig. 2:**
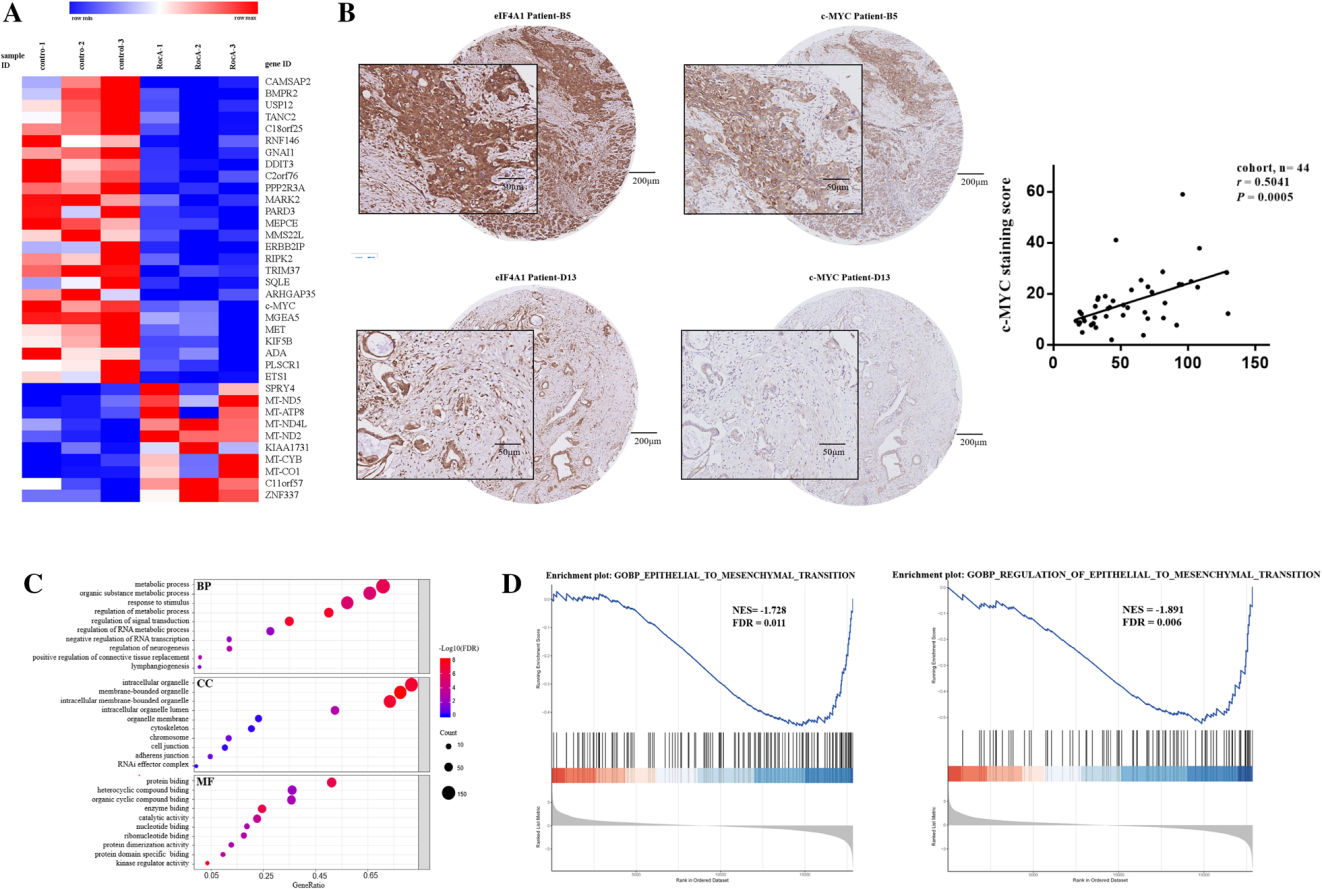
Screening for the target of eIF4A1 in PDAC. **a** c-MYC expression was significantly downregulated (log_2_FC = − 1.05, FDR = 0.00192) in the rocaglate CR-31-B-treated group compared with the control group, ranking in the top 0.6% of all the regulated genes. **b** The expression of eIF4A1 and c-MYC in tumor tissues from the same PDAC patients were analyzed by IHC staining and the results showed that c-MYC is positively correlated with eIF4A1 (n = 44, *r* = 0.5041, *P* = 0.0005, Spearman correlation). **c** The DEPs were enriched in multiple metastasis-associated functions. **d** GSEA was performed with ribosomal sequencing results. Gene enrichment plots showed that the enrichment of EMT-related gene sets was reduced substantially after eIF4A-intervention

To elucidate the mechanism by which eIF4A1 regulates the biological behavior of tumor cells, we searched the GEO database for all ribosome profiling data that included pancreatic cancer cells, and ultimately, dataset GSE120159 was selected for further analysis. The data included 3 Panc-1 cell samples treated with the rocaglate CR-31-B, a small-molecule inhibitor of the eIF4A helicase, and 3 Panc-1 cell samples treated with DMSO. A differential expression analysis using R package DEseq2 identified 179 differentially expressed proteins (DEPs) between the CR-31-B-treated group and the DMSO-treated group, with a *p-*adjusted (FDR) value < 0.05 and |log_2_FC (fold change) |> 1 as the cutoff value. Among these proteins, 128 were downregulated and 51 were upregulated. The heatmap illustrates the proteins with the greatest changes in expression (overexpression and suppression) (Fig. 2a). The expression of c-MYC was significantly downregulated (log_2_FC = − 1.05, FDR = 0.00192) in the CR-31-B-treated group compared with the control group, ranking in the top 0.6% of all the lists of affected genes.

To further screen for the key target of eIF4A that promotes metastasis, we knocked down eIF4A1 expression (eIF4A1 siRNA) and analyzed the alternations of protein profile expression compared to that of the control group. Notably, the protein abundance at 49 kDa, which is the molecular weight of c-MYC remarkably decreased after of eIF4A1 knockdown (Additional file 1: Fig. S1). Importantly, we observed that c-MYC expression was positively correlated with eIF4A1 expression in PDAC and across various cancer types (Fig. 2b; n = 44, *r* = 0.5041, *P* = 0.0005, Spearman correlation; Additional file 2: Fig. S2).

Gene Ontology (GO) analysis using ribosome-sequencing data revealed that the DEPs were significantly enriched in the GO terms for 497 biological processes (BPs), 56 cellular components (CCs), and 48 molecular functions (MFs) (Fig. 2c). We concluded that the DEPs were enriched in functions critical for metastasis, including signal transduction, cytoskeleton, lymph-angiogenesis, and cell junctions. Gene set enrichment analysis (GSEA) performed with ribosomal profiling showed that the enrichment of EMT-related gene sets was reduced significantly after CR-31-B treatment (Fig. 2d). These results indicated that eIF4A1 could target c-MYC to regulate the biological behaviors of pancreatic cancer cells.

### eIF4A1 promoted EMT and metastasis through c-MYC/miR-9 signaling

**Fig. 3:**
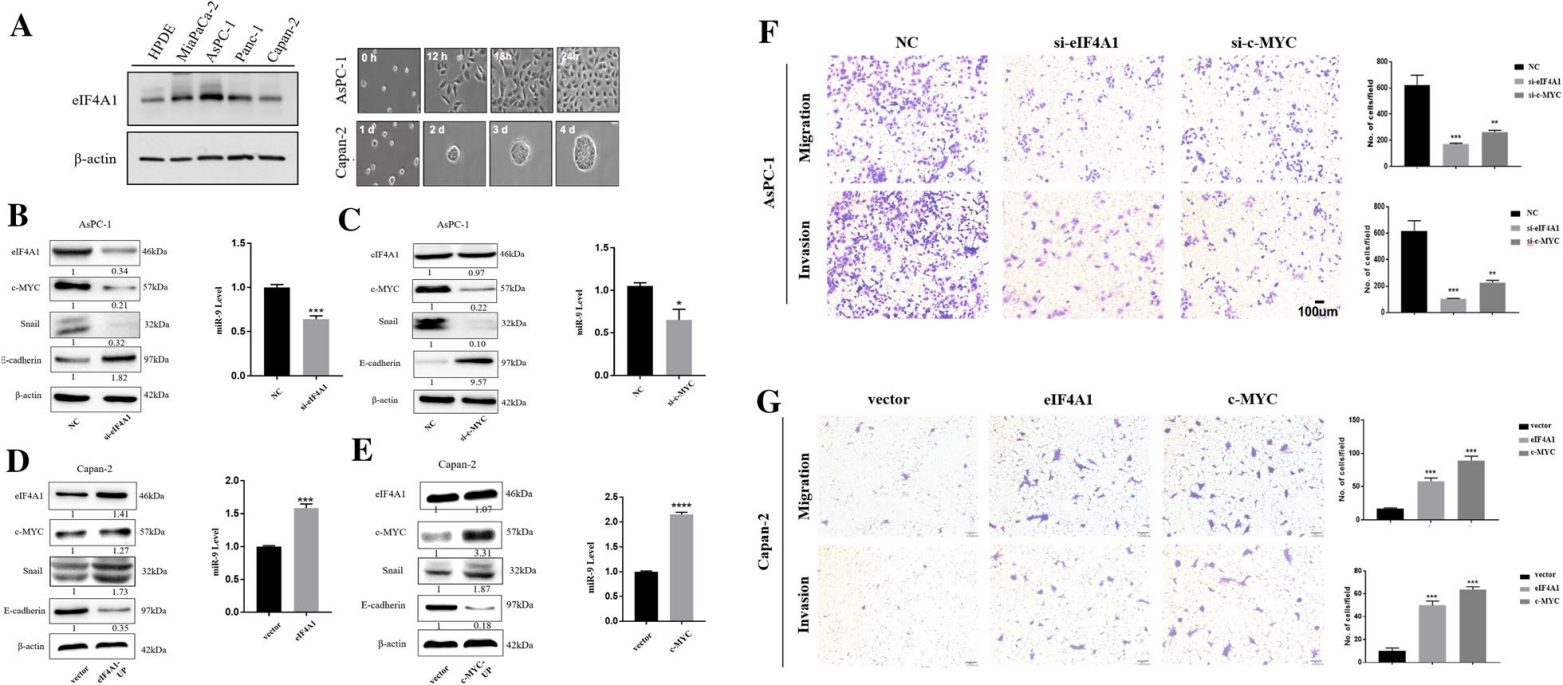
eIF4A1 targeted c-MYC to promote EMT in pancreatic cells. **a** Western blot results show that eIF4A1 was highly expressed in the aggressive AsPC-1 cells and minimally expressed in the indolent Capan-2 cells. **b**, **c** Downregulation of eIF4A1 or c-MYC increased the expression of E-cadherin but decreased the expression of snail and miR-9. Downregulation of eIF4A1 decreased c-MYC expression, but the changes in c-MYC expression did not influence the expression of eIF4A1. **d**, **e** Upregulation of eIF4A1 or c-MYC decreased the expression of E-cadherin but increased the expression of snail and miR-9. Upregulation of eIF4A1 increased c-MYC expression, but the changes in c-MYC expression did not influence the expression of eIF4A1. **f** Knockdown of eIF4A1 or c-MYC impaired the migratory and invasive capabilities of AsPC-1 cells. **g** Overexpression of eIF4A1 or c-MYC enhanced the migratory and invasive capabilities of Capan-2 cells. *P < 0.05, **P < 0.01, ***P < 0.001

Recent studies have demonstrated that c-MYC promotes EMT by upregulating the expression of miR-9, which can competitively bind with E-cadherin encoding sequence of CDH1 and subsequently drive EMT [20, 30]. Before exploring the role of eIF4A1 and c-MYC in the regulatory effect in pancreatic cells, we analyzed eIF4A1 expression in the pancreatic cancer cell lines Panc-1, Capan-2, AsPC-1, and MiaPaca-2, and in the normal pancreatic ductal epithelial cell line HPDE. Western blot analysis showed that eIF4A1 was notably more highly expressed in the aggressive pancreatic cancer cell line AsPC-1 and relatively less expressed in the indolent Capan-2 cell line and normal HPDE cells (Fig. 3a). Therefore, we selected AsPC-1 and Capan-2 for the subsequent experiments. We knocked down eIF4A1 and c-MYC expression in AsPC-1 cells (eIF4A1 siRNA, c-MYC siRNA) and overexpressed eIF4A1 and c-MYC in Capan-2 cells (pcDNA3.1-eIF4A1, pcDNA3.2-myc). Western blot and qRT-PCR showed that eIF4A1 knockdown in AsPC-1 cells decreased the expression of EMT-related genes (c-MYC, snail, and miR-9) and increased the expression of E-cadherin (Fig. 3b). c-MYC knockdown resulted in the same changes in expression (Figure, 3c); however, repressing c-MYC expression did not influence eIF4A1 expression. Accordingly, eIF4A1 and c-MYC overexpression in Capan-2 cells showed expression patterns opposite of those observed in the knockdown experiments (Fig. 3d, e). Transwell migration and invasion assays revealed that AsPC-1 cells with eIF4A1 and c-MYCs knockdown displayed significantly lower migratory and invasive abilities (Fig. 3f), whereas these abilities were significantly increased in eIF4A1 and c-MYC overexpressing Capan-2 cells compared to those in control cells (Fig. 3g). Based on the changes in the expression of EMT-related molecules and the changes in the cells’ migratory and invasive capabilities, these results indicated that eIF4A1 could promote EMT by targeting c-MYC/miR-9 signaling.

### Overexpression of eIF4A1 increased the invasive, migratory, and metastatic capabilities of MYC-knockdown AsPC-1 cells in vitro and in vivo

The relationship between eIF4A1 and c-MYC is not linear, recent studies revealed that overexpression of c-MYC could increase the level of eIF4A1 expression [15, 31]. To further explore the regulatory interactions between eIF4A1 and c-MYC, lentivirus was used to regulate eIFA1 and c-MYC expression in AsPC-1 cells. There were four groups: (1) e-U-M-D sequential regulation group (overexpression of eIF4A1 expression (Lv- eIF4A1) followed by c-MYC knockdown (Lv-sh-c-MYC)); (2) e-U group (overexpression eIF4A1 (Lv- eIF4A1)); (3) M-D group (c-MYC knockdown (Lv-sh-c-MYC)); (4): vector group (Lv-sh-control).

**Fig. 4:**
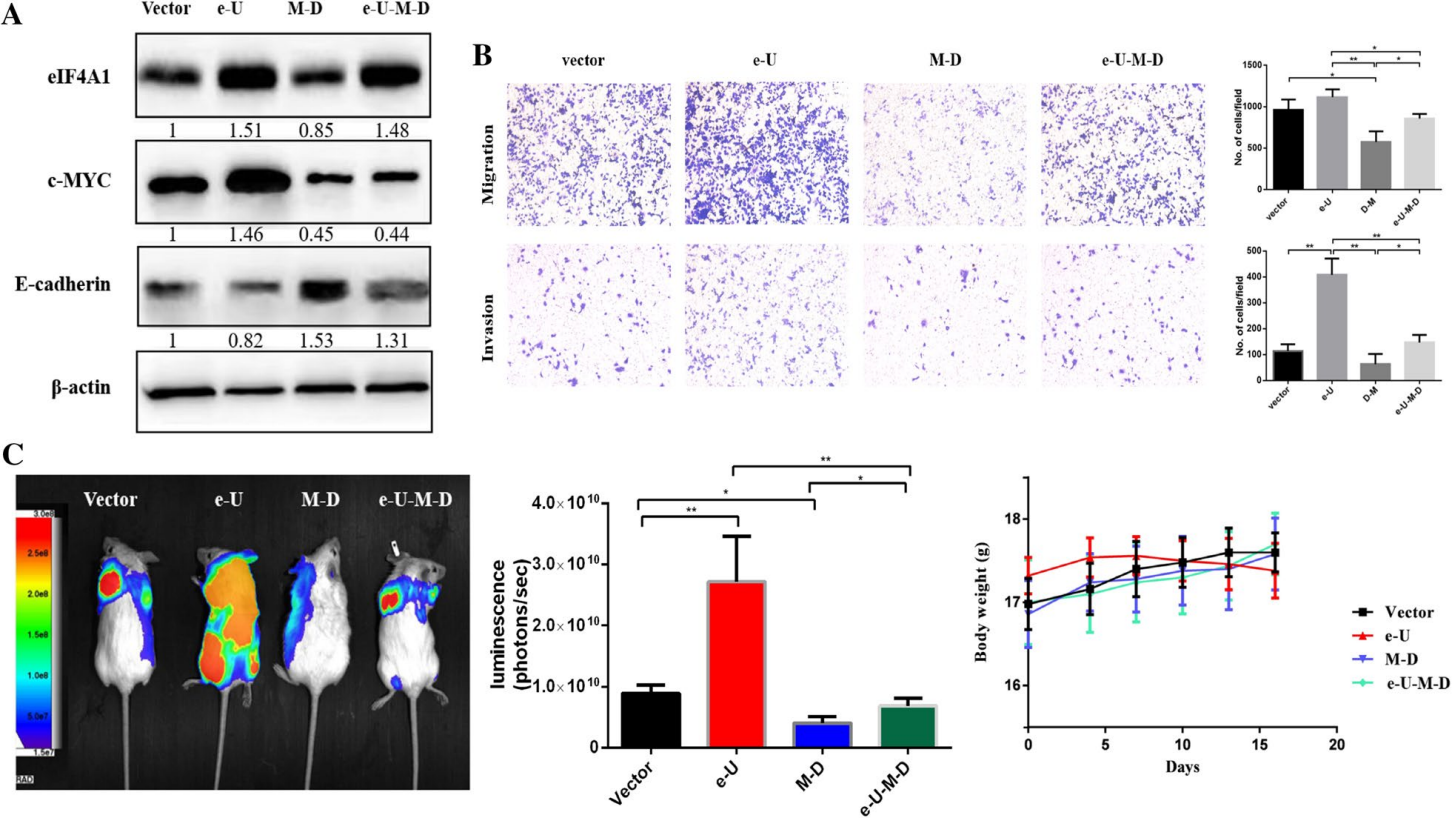
Sequential regulation of eIF4A1 and c-MYC expression in vitro and in vivo*.* **a** E-cadherin expression in AsPC-1 cells in the e-U-M-D group was between that in AsPC-1 cells in the e-U group and that in AsPC-1 cells in the M-D group. **b** The migratory and invasive capabilities of e-U-M-D AsPC-1 cells were significantly stronger than those of M-D cells. **c** The metastatic capabilities of e-U-M-D cells were between those of e-U and M-D cells in vivo. *P < 0.05, **P < 0.01, ***P < 0.001

Western blots showed that E-cadherin expression was significantly decreased in e-U AsPC-1 cells and significantly increased in the M-D AsPC-1 cells. The E-cadherin expression in e-U-M-D AsPC-1 cells was between that in the e-U and M-D cells but was still higher than the level in the vector group cells (Fig. 4a). Consistent with the trends of western blot results, Transwell migration and invasion assays showed that the migratory and invasive abilities of e-U-M-D cells were superior to those of the M-D cells but inferior to those of the vector and e-U cells (Fig. 4b). In vivo, the metastatic potentials of these cells were examined using a mouse metastasis model via caudal vein injection. The results revealed that the luminescence intensity of e-U-M-D cells was significantly higher than M-D group cells (1.008e + 10 vs. 5.387e + 9, *P* = 0.0349), accordingly weaker to the e-U group cells (1.008e_+_ 10 vs. 2.410e_+_ 10, *P* = 0.2369) (Fig. 4c). Collectively, the in vitro and in vivo results revealed that the level of EMT and metastatic capabilities of e-U-M-D cells were between those of the e-U cells and M-D cells. These results indicated that overexpression of eIF4A1 expression could attenuate the inhibition of MYC-downregulated pancreatic cancer cells of EMT and metastasis.

### RocA alone was not inferior to RocA plus Mycro3 joint intervention inhibiting EMT and metastasis in vitro and in vivo

Our previous studies demonstrated that c-MYC was not the only target of eIF4A1 to promote EMT and metastasis. When c-MYC is repressed, eIF4A1 might exert a pro-EMT effect through other pathways to compensate for this inhibition. Considering the complex feedback relationship between eIF4A1 and c-MYC, we implemented a combination treatment comprising RocA (an eIF4A1 inhibitor) and Mycro3 (a c-MYC inhibitor) to explore whether the combination is superior to either monotherapy.

**Fig. 5:**
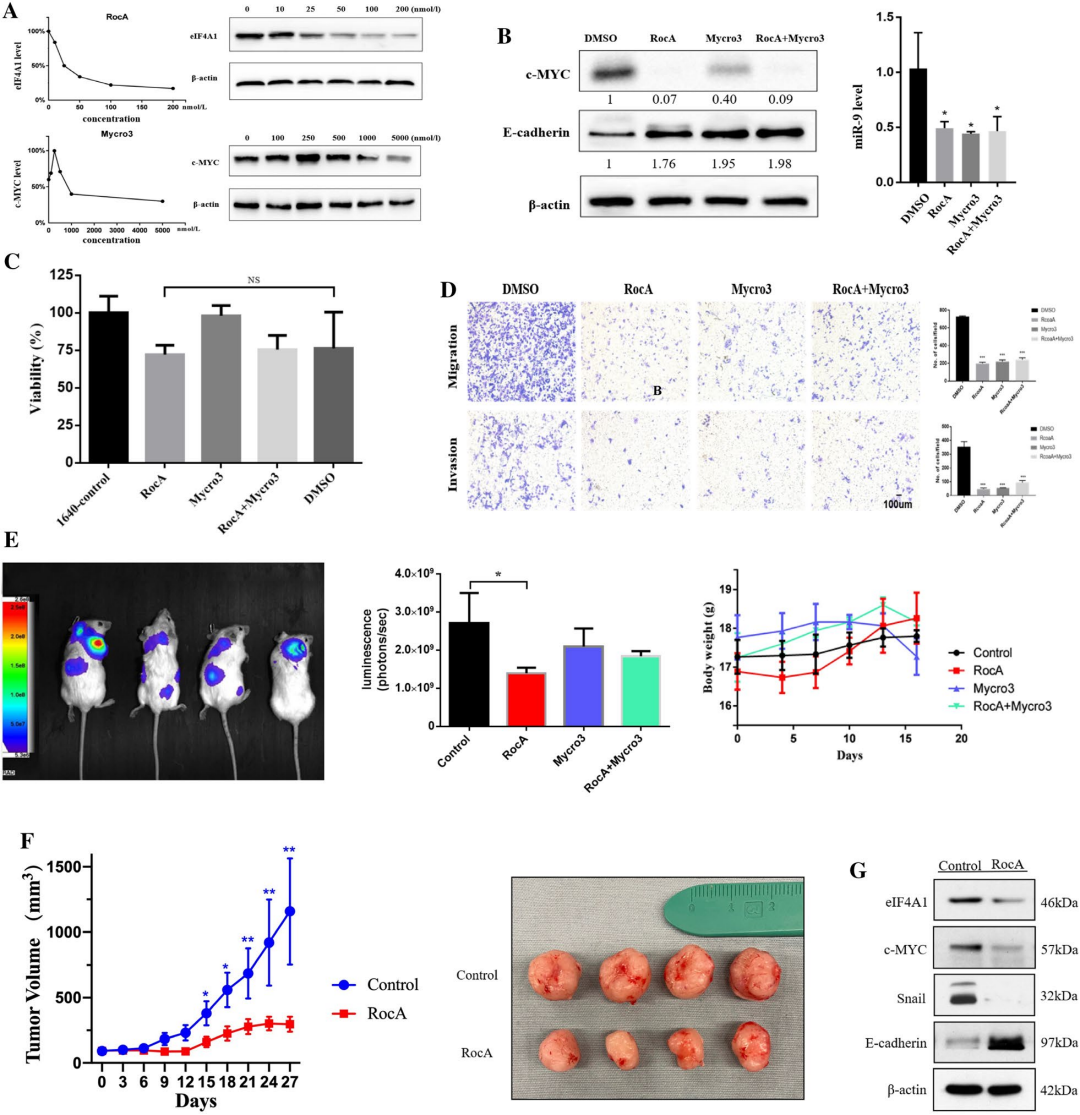
RocA and Mycro3 suppressed EMT and metastasis in pancreatic cancer cells in vitro and in vivo*.* **a** The suitable concentrations for treatment were initially determined to be 100 nmol/L (RocA) and 5000 nmol/L (Mycro3). **b** Treatment with 100 nmol/L RocA and 5000 nmol/L Mycro3, and either alone or in combination significantly inhibited the expression of miR-9 and c-MYC in AsPC-1 cells. **c** AsPC-1 cells were treated with DMSO, RocA (100 nM), Mycro3 (5000 nM), and combination treatment for 4 h and evaluated by CCK8 assay. It showed that there were no significant differences of the death ratio among each treatment group. **d** Treatment with 100 nmol/L RocA and 5000 nmol/L Mycro3 either alone or in combination significantly decreased the migratory and invasive capabilities of AsPC-1 cells. There were no significant differences among the treatment groups. **e** RocA significantly decreased the metastasis level of AsPC-1 cells in vivo whereas Mycro3 and the combination treatment did not. **f** RocA significantly suppressed subcutaneous tumor xenograft growth. **g** Western blot results showed that RocA notably decreased the expression of eIF4A1, c-MYC, and snail, but increased the expression of E-cadherin in subcutaneous tumor grafts. *P < 0.05, **P < 0.01, ***P < 0.001, NS: no statistical significance

To select the optimal drug concentration, we conducted a series of concentration gradient experiments. The western blot results showed that eIF4A1 expression decreased significantly in response to 100 nM RocA and that c-MYC expression decreased significantly in response to 5000 nM Mycro3 (Fig. 5a). Crystal precipitates formed when the concentrations increased beyond the value in subsequent experiments; thus, we selected 100 nM RocA and 5000 nM Mycro3 in subsequent experiments. We established 4 treatment groups [(1) RocA + Mycro3; (2) RocA; (3) Myro3; (4) DMSO control] and compared their safety and efficacy. Western blots and qRT-PCR showed that all 3 intervention methods significantly increased E-cadherin expression and decreased c-MYC expression in AsPC-1 cells (Fig. 5b). Compared with the DMSO treated cells, CCK8 assay showed that pretreatment with RocA and Mycro3 either alone or in combination did not affect the death ratio of AsPC-1 cells (Fig. 5c). However, all 3 intervention methods remarkably decreased the migratory and invasive abilities and methods decreased the EMT level of pancreatic cancer cells compared with the control group (Fig. 5d; Additional file 3: Fig. S3). However, there was no statistically significant difference among the 3 groups.

To examine the efficiency and safety of the treatments in vivo, we injected mice with AsPC-1 cells via caudal vein. We found that the mortality rates of the RocA group (40.0%) and combination group (62.5%) were relatively high. Evaluation of mice after sacrifice showed multiple cases presenting nonocclusive intestinal dilation. Considering the possible intolerance due to the high dose and frequency of RocA administered, we decreased the dosage from 5 mg/kg/d qd to 2.5 mg/kg/d once on alternate days (still administered via intraperitoneal injection), while the dosage of Mycro3 remains 100 mg/kg/d qd (administered intragastrically). The mice tolerated the modified regimen better without more deaths. The luminescence intensity from the RocA group was significantly weaker than that of the control group (1.393e + 9 vs. 2.707e + 9, *P* = 0.0474). However, there were no significant differences in luminescence intensity between the control and both the Myro3 and combination groups (Fig. 5e). Taken together, these data demonstrated that the modified of RocA monotherapy exerted an obvious antimetastatic effect, superior to Mycro3 or the combination treatment.

To further evaluate the efficacy of RocA, we used a subcutaneous xenograft nude mouse model. Two-way ANOVA indicated that the tumor volumes of the RocA group were significantly smaller than those of the control group (*P* < 0.0001) (Fig. 5f) which suggested that RocA notably suppressed tumor growth. Western blot analysis showed that RocA markedly decreased the expression of eIF4a1, c-MYC, and snail and increased the expression of E-cadherin (Fig. 5g) in vivo.

## Discussion

Various oncogenes associated with PDAC, including KRAS and its downstream kinases cannot be targeted because of their specific molecular structures [23, 32]. However, targeting translation has been an alternative approach for cancer treatment. Protein production in PDAC was verified to be hyperactive both in vivo and in organoids [33]. eIF4A, which participates in the assembly of eIF4F, is the nexus for translational regulation [13, 34]. Unlike KRAS, eIF4A can be targeted, and the curative effects of targeting eIF4A have been validated in several hematological malignancies [25, 35], positioning this molecule as an alternative treatment for PDAC. Recent studies revealed that eIF4A1 inhibition could significantly repress the tumor growth by remodeling metabolism and DNA replication [36, 37]. Here, we present a novel strategy for inhibiting EMT and metastasis in pancreatic cancer cells via c-MYC/miR-9 signaling.

Our previous study demonstrated that targeting eIF4A could significantly decrease the lung metastasis of pancreatic cancer cells in vivo [26]. Here we further elucidated the mechanism by which eIF4A promotes EMT and metastasis and verified that eIF4A1 was correlated with a worse prognosis. Considering the complex long-sequence 5′-UTR structure of c-MYC mRNA, we showed that the regulation of c-MYC expression was highly dependent on eIF4A. Depletion of eIF4A1 significantly decreased the expression levels of c-MYC and EMT markers in vitro. Overexpression of eIF4A1 induced a decrease in E-cadherin expression through the c-MYC/miR-9 axis. Furthermore, depletion of either eIF4A1 or c-MYC significantly decreased the EMT level and metastasis in vitro and in vivo, and vice versa. However, regulating translation is extremely complicated. c-MYC is likely not the only target for eIF4A1 to promote EMT and metastasis and it was reported that c-MYC overexpression could further upregulate eIF4A1 [31, 38]. In this study, we found that eIF4A1 overexpression rescued the diminished EMT and metastasis capabilities of c-MYC knockdown pancreatic cancer cells in vitro and in vivo. Nevertheless, the compensation for c-MYC depletion was incomplete, as the metastatic capabilities of e-U-M-D cells were significantly weaker than those of e-U cells whereas stronger than those of M-D cells. This result indicated that even if there are other targets of eIF4A1, c-MYC still plays a vital role in eIF4A-mediated EMT and metastasis.

Similar to KRAS, c-MYC has traditionally been deemed an “undruggable” target. However, Mycro3 is a novel anti-MYC compound inhibits c-MYC activity through MYC dimerization [23, 39]. As previously mentioned, the relationship between eIF4A and c-MYC was not simply unidirectional; thus, simultaneously targeting eIF4A and c-MYC was employed, and the efficacy and safety of this regimen were evaluated. Our work demonstrated that RocA or Mycro3 either alone or in combination notably repressed the EMT level in vitro. However, the combination treatment did not show any superiority to the monotherapies. Similar to the in vitro results, RocA was the only reagent that exerted a significant antimetastatic effect in vivo among the two monotherapy and combination treatment. The reason behind this result is still unknown, but we can speculate from preparatory experiments that combining RocA and Mycro3 increased the risk of death, as the mice were not tolerant of the original dose. The dose of RocA in previous studies ranged from 1.4 mg/kg once on an alternate day to 5 mg/kg daily for cancer treatment [26, 35, 40–45], which was quite extensive. Further toxicity analysis is required to elucidate the most accurate treatment dose selection. All previous studies using RocA for pancreatic cancer treatment was administered 5 mg/kg/d (26, 41). However, our study showed that mid-dose 2.5 mg/kg every other day RocA monotherapy also dramatically suppressed eIF4A-mediated pancreatic cancer cell metastasis.

## Conclusions

**Fig. 6:**
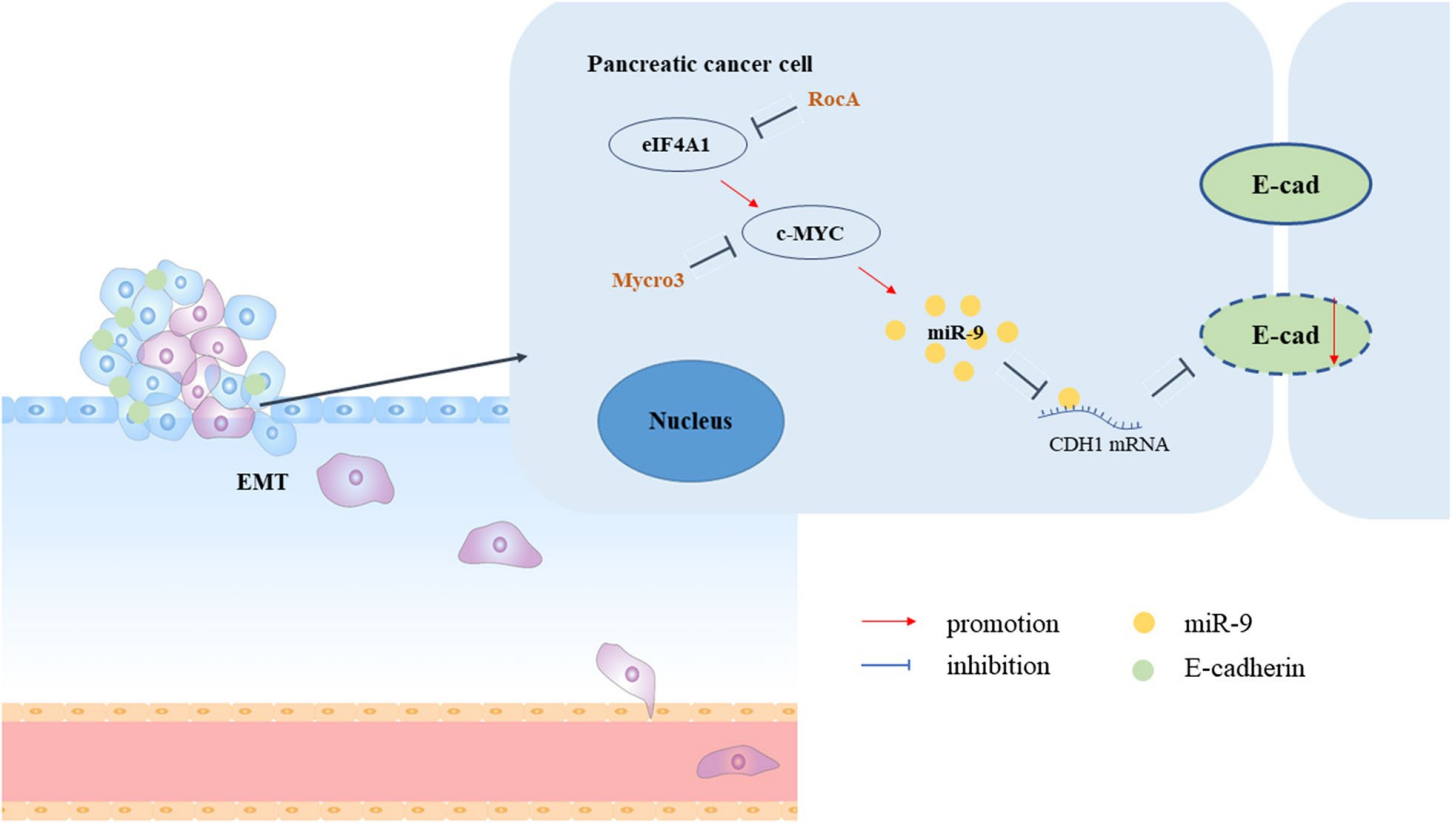
eIF4A1 promotes EMT in pancreatic cancer cells via c-MYC/miR-9 signaling

Collectively, we demonstrated that eIF4A1 overexpression downregulated E-cadherin expression through the c-MYC/miR-9 axis (Fig. 6), which promoted EMT and the metastasis of pancreatic cancer cells in vitro and in vivo. In addition, ectopic expression of eIF4A1 partially rescued the inhibition of EMT and metastasis mediated by c-MYC-depletion. Moreover, RocA monotherapy was superior to the combination treatment at the current dose, possibly due to intolerance. Our data indicated that eIF4A1 is a satisfactory biomarker for PDAC prognosis, and targeting eIF4A1 provides a promising therapeutic strategy for overcoming PDAC metastasis.

## Supplementary Information

**Additional file 1: Fig. S1.** SDS-PAGE of eIF4A1-knockdown AsPC-1 cells. The protein abundance at 49 kDa (the molecular weight of c-MYC) remarkably decreased after the knockdown of eIF4A1.

**Additional file 2: Fig. S2.** Analysis of the correlation of eIF4A1 and c-MYC using data from TCGA and GTEx databases. (a). The expression of eIF4A1 and c-MYC in tumor tissues and normal tissues of PDAC patients were analyzed. c-MYC is positively correlated with eIF4A1 (n = 350, *r* = 0.475, *P* = 2.26e-16, Spearman correlation). (b). c-MYC and eIF4A1 are positively correlated in most cancer types including PDAC.

**Additional file 3: Fig. S3.** Fluorescence in situ hybridization (FISH) assay showed that treatment with 100 nM RocA for 12 h significantly decreased the miR-9 expression in AsPC-1 cells.

## Data Availability

The datasets used and/or analyzed during the current study available from the corresponding author on reasonable request.

## Abbreviations

PDAC: Pancreatic ductal adenocarcinoma
eIF: Eukaryotic translation initiation factor
EMT: Epithelial mesenchymal-transition
RocA: Rocaglamide
TCGA: The Cancer Genome Atlas
GTEx: Genome tissue expression
5’-UTR: 5’-Untranslated region
FC: Fold change
FDR: False discovery rate
DEP: Differential expressed protein
