# Supplementary figures and images for: Targeted intervention of eIF4A1 inhibits EMT and metastasis of pancreatic cancer cells via c-MYC/miR-9 signaling
Source: Cancer Cell Int. 2021 Dec 14;21:670. doi: 10.1186/s12935-021-02390-0 (PMC8672469; doi:10.1186/s12935-021-02390-0)

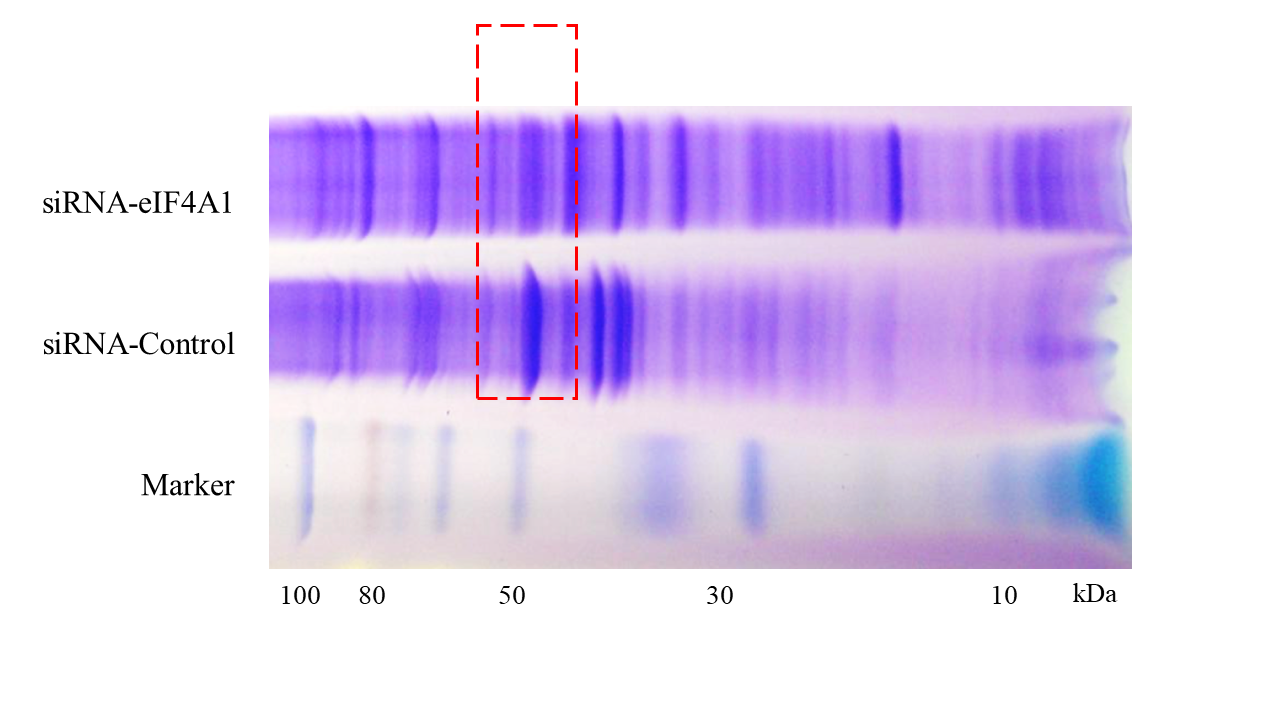

Supplement: Supplementary file 1 — Additional file 1: Fig. S1. SDS-PAGE of eIF4A1-knockdown AsPC-1 cells. The protein abundance at 49 kDa (the molecular weight of c-MYC) remarkably decreased after the knockdown of eIF4A1. [file 12935_2021_2390_MOESM1_ESM.tif]

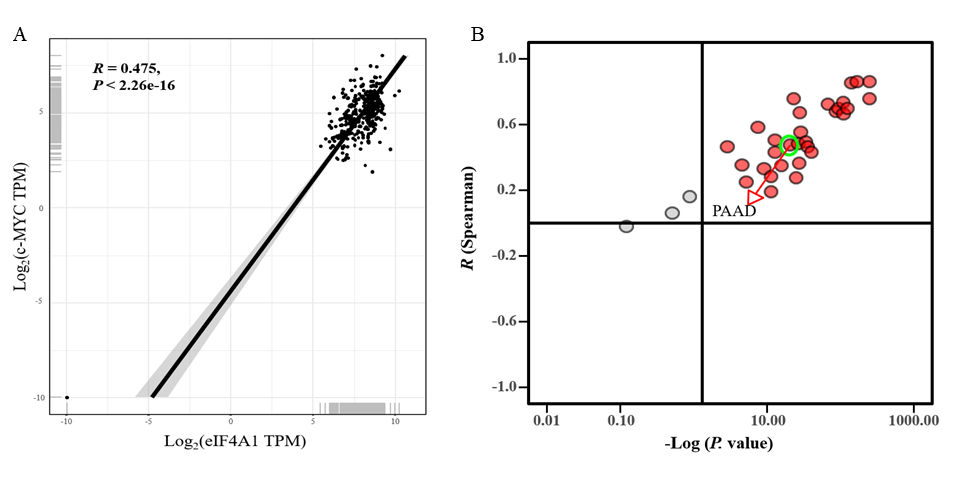

Supplement: Supplementary file 2 — Additional file 2: Fig. S2. Analysis of the correlation of eIF4A1 and c-MYC using data from TCGA and GTEx databases. (a). The expression of eIF4A1 and c-MYC in tumor tissues and normal tissues of PDAC patients were analyzed. c-MYC is positively correlated with eIF4A1 (n = 350, r = 0.475, P = 2.26e-16, Spearman correlation). (b). c-MYC and eIF4A1 are positively correlated in most cancer types including PDAC. [file 12935_2021_2390_MOESM2_ESM.tif]

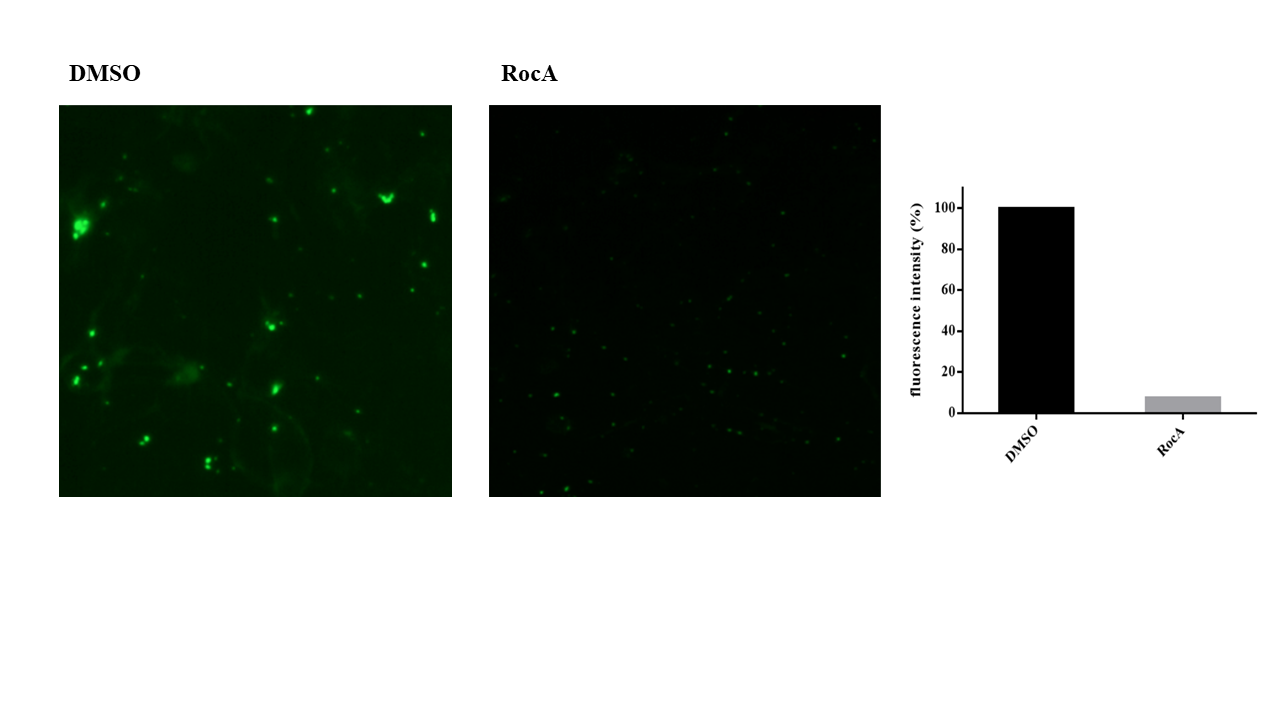

Supplement: Supplementary file 3 — Additional file 3: Fig. S3. Fluorescence in situ hybridization (FISH) assay showed that treatment with 100 nM RocA for 12 h significantly decreased the miR-9 expression in AsPC-1 cells. [file 12935_2021_2390_MOESM3_ESM.tif]
